# Three *FT* and multiple *CEN* and *BFT* genes regulate maturity, flowering, and vegetative phenology in kiwifruit

**DOI:** 10.1093/jxb/erx044

**Published:** 2017-03-27

**Authors:** Charlotte Voogd, Lara A. Brian, Tianchi Wang, Andrew C. Allan, Erika Varkonyi-Gasic

**Affiliations:** 1 The New Zealand Institute for Plant & Food Research Limited (Plant & Food Research) Mt Albert, Private Bag 92169, Auckland Mail Centre, Auckland 1142, New Zealand; 2 School of Biological Sciences, University of Auckland, Private Bag 92019, Auckland 1142, New Zealand

**Keywords:** *Actinidia chinensis*, BFT, CEN, development, dormancy, flowering, FT, kiwifruit.

## Abstract

Kiwifruit is a woody perennial horticultural crop, characterized by excessive vegetative vigor, prolonged juvenility, and low productivity. To understand the molecular factors controlling flowering and winter dormancy, here we identify and characterize the kiwifruit PEBP (phosphatidylethanolamine-binding protein) gene family. Five *CEN*-like and three *BFT*-like genes are differentially expressed and act as functionally conserved floral repressors, while two *MFT*-like genes have no impact on flowering time. *FT*-like genes are differentially expressed, with *AcFT1* confined to shoot tip and *AcFT2* to mature leaves. Both act as potent activators of flowering, but expression of *AcFT2* in Arabidopsis resulted in a greater impact on plant morphology than that of *AcFT1*. Constitutive expression of either construct in kiwifruit promoted *in vitro* flowering, but *AcFT2* displayed a greater flowering activation efficiency than *AcFT1*, leading to immediate floral transition and restriction of leaf development. Both leaf and flower differentiation were observed in *AcFT1* kiwifruit lines. Sequential activation of specific PEBP genes in axillary shoot buds during growth and dormancy cycles indicated specific roles in regulation of kiwifruit vegetative and reproductive phenologies. *AcCEN* and *AcCEN4* marked active growth, *AcBFT2* was associated with suppression of latent bud growth during winter, and only *AcFT* was activated after cold accumulation and dormancy release.

## Highlight

Sequential action of kiwifruit *CEN*, *BFT*, and *FT* genes regulates growth, dormancy, and flowering. Two differentially expressed *FT* genes have different impacts on flowering and plant morphology.

## Introduction

The phosphatidylethanolamine-binding proteins (PEBPs), initially identified in animals as Raf-1 kinase inhibitors (Yeung *et al.*, 1999), provide a potential to fine-tune developmental regulatory mechanisms and present opportunities for improvement of agriculturally important traits (Jung and Müller, 2009; Zhang *et al.*, 2010; Yeoh *et al.*, 2011; Iwata *et al.*, 2012; Putterill *et al.*, 2013; Koskela *et al.*, 2016). In angiosperms, this family consists of three phylogenetically distinct groups: the FLOWERING LOCUS T (FT)-like proteins (Kardailsky *et al.*, 1999; Kobayashi *et al.*, 1999), the TERMINAL FLOWER1 (TFL1)-like and CENTRORADIALIS (CEN)-like proteins (Bradley *et al.*, 1996, 1997; Ohshima *et al.*, 1997), and the MOTHER OF FT AND TFL1 (MFT)-like proteins (Mimida *et al.*, 2001; Yoo *et al.*, 2004). In addition, the BROTHER OF FT AND TFL1 (BFT)-like proteins (Yoo *et al.*, 2010) represent a separate subclade within the CEN group.

*FT*- and *TFL1*/*CEN*-like genes have conserved roles in regulation of flowering, and the ancestral *MFT*-like genes (Hedman *et al.*, 2009; Karlgren *et al.*, 2011) have been associated with seed germination (Xi *et al.*, 2010; Footitt *et al.*, 2011; Nakamura *et al.*, 2011). FT-like proteins can act as florigen (Chailakhyan, 1968; Zeevaart, 2008), moving in the phloem from leaves to the shoot apex in a highly regulated manner (reviewed in Putterill and Varkonyi-Gasic, 2016) to promote flowering. In contrast, *TFL1*/*CEN*-like proteins repress flowering and maintain the inflorescence meristem (Shannon and Meeks-Wagner, 1991; Ratcliffe *et al.*, 1998, 1999; Conti and Bradley, 2007). This antagonistic activity (Shalit *et al.*, 2009; Lifschitz *et al.*, 2014) involves interaction with a common partner FD (Pnueli *et al.*, 2001; Abe *et al.*, 2005; Wigge *et al.*, 2005; Danilevskaya *et al.*, 2010; Hanano and Goto, 2011; Randoux *et al.*, 2014) and is conferred by a divergent external loop and specific amino acids in positions critical for FT function (Hanzawa *et al.*, 2005; Ahn *et al.*, 2006). Other changes resulting in altered surface charge can affect function (Ho and Weigel, 2014), and some FT-like proteins act as repressors of flowering (Pin *et al.*, 2010; Harig *et al.*, 2012; Cao *et al.*, 2016). In many plant species, numerous *FT*-like genes arose from gene duplication events (Pin and Nilsson, 2012), but only one or two act in flowering control, and *FT*-like roles besides flowering, such as vegetative growth and storage organ differentiation, have been reported (Hsu *et al.*, 2011; Navarro *et al.*, 2011; Lee *et al.*, 2013), confirming a key role for PEBPs in co-ordination of general growth and development.

Many aspects of PEBP biology remain less well understood in woody perennial plants (Putterill and Varkonyi-Gasic, 2016). Recent studies of the most advanced woody perennial model poplar (*Populus* spp.) revealed conservation between molecular regulation of flowering in annual plants and seasonal phenology in trees (Ding and Nilsson, 2016). In addition to regulation of flowering, poplar *FT*- and *TFL1*/*CEN*-like genes are implicated in the regulation of vegetative to reproductive transition, growth and dormancy cycles, and the co-existence of vegetative and floral meristems on the same shoot (Böhlenius *et al.*, 2006; Hsu *et al.*, 2006, 2011; Rinne *et al.*, 2011). Similarly, *TFL1* is a key regulator of perennial growth and seasonality of flowering in strawberry and rose (Iwata *et al.*, 2012; Koskela *et al.*, 2012, 2016; Randoux *et al.*, 2014; Rantanen *et al.*, 2015), down-regulation of *TFL1*-like genes accelerated flowering in apple and pear (Kotoda *et al.*, 2006; Freiman *et al.*, 2012; Yamagishi *et al.*, 2016), ectopic overexpression of a *CEN* homolog suppressed flowering in kiwifruit (Varkonyi-Gasic *et al.*, 2013), and a connection of *TFL1* accumulation with biennial bearing in apple has been established (Haberman *et al.*, 2016).

On the other hand, constitutive or inducible expression of Arabidopsis or poplar *FT* genes has been attempted and used with some success to accelerate breeding (Srinivasan *et al.*, 2012; Wenzel *et al.*, 2013), although many questions regarding the *FT* gene, promoter, and feasibility of the approach remain (Zhang *et al.*, 2010). Remarkably, only a handful of endogenous *FT* genes have been functionally characterized by transgenic approaches in woody perennial species; expression under a strong constitutive promoter resulted in extremely early flowering in trifoliate orange, poplar, apple, and blueberry (Endo *et al.*, 2005; Böhlenius *et al.*, 2006; Hsu *et al.*, 2006; Kotoda *et al.*, 2010; Yamagishi *et al.*, 2011, 2014; Song *et al.*, 2013), and only one example of graft-transmissible early flowering has been reported in a small woody shrub, Barbados nut (*Jatropha*) (Ye *et al.*, 2014). Even less is known about functional diversification of *FT* genes in woody perennials. Ectopic expression implied distinctive roles in reproductive and vegetative growth for poplar *FT* paralogs (Hsu *et al.*, 2011), not yet confirmed by paralog-specific silencing; expression of only one of two *FT* genes in apple and one of three *FT* genes in citrus has been associated with transition to flowering (Nishikawa *et al.*, 2007; Kotoda *et al.*, 2010), and expression of a single grape *FT* gene could be detected irrespective of flowering (Sreekantan and Thomas, 2006; Carmona *et al.*, 2007). Furthermore, some *FT* genes failed to induce early flowering in woody perennials, despite their ability to promote flowering in model annuals, while affecting dormancy and leaf senescence in apple (Freiman *et al.*, 2015) or shoot vigor in kiwifruit (Varkonyi-Gasic *et al.*, 2013).

Although the ability to regenerate transgenic woody perennials efficiently remains an obstacle, development of new technologies to induce gene-specific biallelic homozygous mutations in trees (Fan *et al.*, 2015) and specifically manipulate members of the PEBP family holds great promise in accelerating the breeding process and improving the architecture and productivity of woody perennials of horticultural importance. Detailed functional characterization of PEBP genes is the necessary first step to improve selection of gene candidates likely to have a key role in maturation and flowering.

Kiwifruit (*Actinidia*) is one of the most recently domesticated horticultural crops, with a short history of breeding (Datson and Ferguson, 2011), limited genetic resources, and little understanding of molecular regulation or the genetic diversity in flowering responses to internal and environmental signals. Commercial kiwifruit cultivars belong to closely related *A. chinensis* and *A. deliciosa*, which, together with other members of the *Actinidia* genus, are woody perennial vines from the order Ericales in the asterid lineage, with features of development specific to temperate woody perennials, including prolonged juvenility, growth spread over two seasons (Brundell, 1975*a*, *b*), and a period of winter chilling required to resume growth after winter dormancy (Brundell, 1976). Excessive vegetative growth and a small number of flowers, resulting in low fruit yield and high orchard costs, are key targets in kiwifruit improvement strategies. Flower differentiation occurs during budbreak in spring, from pre-established but undifferentiated overwintering primordia (Walton *et al.*, 1997, 2001; Varkonyi-Gasic *et al.*, 2011). Inflorescences develop in the lower leaf axils of the shoots that emerge after sufficient winter chilling, and photoperiod has no clear role in regulation of flowering in spring (Snelgar *et al.*, 2007).

An *FT* and a *CEN* gene have been described in kiwifruit previously (Varkonyi-Gasic *et al.*, 2013), but *AcFT* failed to induce precocious flowering in transgenic kiwifruit and it remained unclear if other PEBP genes might be involved in regulation of kiwifruit maturity, architecture, and phenology. The completion of the draft genome sequence of *A. chinensis* (Huang *et al.*, 2013) provided the opportunity to identify additional sequences belonging to the kiwifruit PEBP family. In this study, we identified 13 *Actinidia* PEBP genes, five belonging to the *TFL1*/*CEN* clade, three each belonging to the *BFT* and *FT* clade, and two belonging to the *MFT* clade. All genes with the exception of the previously characterized *AcFT* and *AcCEN* (Varkonyi-Gasic *et al.*, 2013) were characterized here using a combination of expression analysis and functional studies in transgenic Arabidopsis. Two of the previously undescribed *FT*-like genes were further characterized in more detail and their role in activation of flowering was evaluated in transgenic kiwifruit. The information provided help to better understand the diversity and biological function of the PEBP family in woody perennial plants and identifies candidate targets for improvement of kiwifruit architecture, flowering time, and plant determinacy.

## Materials and methods

### Plant materials

Kiwifruit plant material was sampled from female kiwifruit cultivars, ‘Hort16A’ (*Actinidia chinensis* Planch., recently classified as *A. chinensis* Planch. var. *chinensis*) and ‘Hayward’ [*A. deliciosa* (A. Chev.) C.F. Liang et A.R. Ferguson, recently classified as *A. chinensis* var. *deliciosa* (A. Chev.) A. Chev.]. Sampling for gene expression analysis was described previously (Wu *et al.*, 2012; Varkonyi-Gasic *et al.*, 2013). Briefly, root, stem, leaf, flower, and fruit tissue were collected from mature plants grown in the Plant & Food Research orchard near Kerikeri, New Zealand in the spring and summer season of 2005–2006, basal and distal leaves from mature ‘Hort16A’ plants grown in the Plant & Food Research orchard near Te Puke in spring of 2010, and axillary bud samples from field-grown ‘Hayward’ plants grown in a commercial orchard near Hamilton, New Zealand during the 1995–1996 season. Amplification of full-length coding sequences was performed on a set of ‘Hort16A’ cDNA described in Ledger *et al.* (2010). Additional leaf samples (L1–L8) were collected from 1-year-old ‘Hort16A’ plants grown in the Plant & Food Research glasshouse in Auckland, New Zealand in spring 2013. Axillary bud samples used in RNA-seq experiments were collected at monthly intervals from *A. chinensis* plants grown in the Plant & Food Research orchard near Te Puke, New Zealand in the 2014–2015 season. Terminal buds were sampled from the same plants in January and March 2015. Three plants (biological replicates) were used and ~10 buds were sampled at each time point, with the exception of the March terminal bud sample, where only two plants were available. Sampling of tissues was performed at mid-day to avoid variation.

### Isolation of the genes and vector construction

Gene-specific oligonucleotide primers were designed based on available sequence data (Huang *et al.*, 2013) (see Supplementary Table 1 at *JXB* online). Full-length coding sequences were amplified from *A. chinensis* ‘Hort16A’ cDNA, cloned into the pUC19 vector, and verified by sequence analysis (GenBank accession nos KX611594–KX611604). After addition of attB sites by PCR, each gene was recombined using Gateway into pDONR221, verified by sequence analysis, then recombined into pHEX2 (Hellens *et al.*, 2005), placing each cDNA between the *Cauliflower mosaic virus* (CaMV) *35S* promoter and the *ocs* 3' transcriptional terminator. The modified *AcFT1* and *AcFT2* sequences with added attB sites were synthesized by GenScript (http://www.genscript.com) and recombined into pHEX2 as above. For *SUC2:AcFT1* and *SUC2:AcFT2* constructs, the cDNAs were re-amplified to include appropriate restriction sites and cloned as *Spe*I–*Xba*I fragments into pSAK778-SUC2 to replace *AcFT* (Varkonyi-Gasic *et al.*, 2013). The *35S:GUS* vector control was constructed previously (Drummond *et al.*, 2009). All resulting plasmids were transformed into *Agrobacterium tumefaciens* strain GV3101 by electroporation. *35S:AcFT1* and *35S:AcFT2* were also transformed into *A. tumefaciens* strain EHA105 by electroporation.

### Phylogeny

Predicted protein sequence alignment and phylogenetic analyses were conducted using Vector NTI (Invitrogen) CLUSTAL W, and phylogeny was constructed using a minimum evolution phylogeny test and 1000 bootstrap replicates in MEGA version 5.2 (Tamura *et al.*, 2011).

### Plant transformation and growth

*Agrobacterium tumefaciens*-mediated Arabidopsis transformation was performed as described (Clough and Bent, 1998; Martinez-Trujillo *et al.*, 2004), using constructs in *A. tumefaciens* strain GV3101. Seeds of transgenic plants were selected on half-strength Murashige and Skoog (1/2 MS) medium supplemented with kanamycin and placed in a growth room under a long-day (LD, 21 °C, 16/8 h light/dark) or short-day (SD, 21 °C, 8/16 h light/dark) regime. Plants were grown in soil using a standard potting mix. Transformation of *A. chinensis* ‘Hort16A’ was as previously described (Wang *et al.*, 2007; Voogd *et al.*, 2015), using *A. tumefaciens* strain EHA105.

### RNA extraction and expression studies

Total RNA from Arabidopsis was isolated using the Trizol reagent (Invitrogen) and from kiwifruit using the Spectrum Plant Total RNA Kit (Sigma-Aldrich, St. Louis, MO, USA). Reverse transcription was performed using the QuantiTect Reverse Transcription Kit (Qiagen). Quantifications using real-time PCR were performed with the FastStart DNA Master SYBR Green I mix (Roche Diagnostics, Mannheim, Germany) using the LightCycler 1.5 instrument and the LightCycler Software version 4 (Roche Diagnostics). Oligonucleotide primers (Supplementary Table S1) were designed to produce amplification products of 100–150 nucleotides. The specificity of primer pairs was confirmed by melting curve analysis of PCR products and agarose gel electrophoresis followed by sequence analysis. The expression was normalized to *Actinidia ACTIN* (GenBank accession no. FG403300) or Arabidopsis *ACT2* (At3g18780).

The axillary bud sequencing libraries were constructed according to the TruSeq RNA sample preparation guide (Illumina, San Diego, CA, USA) and subsequently sequenced by a HiSeq 2500 Sequencing System (Illumina) at Australia Genomics Resource Facility (Brisbane, Australia), obtaining paired-reads of 125 bp. Raw sequence data in FASTQ format were filtered to remove the adaptors, low-quality reads, and reads containing ambiguous nucleotides, resulting in an average of 13.8 million reads per library. As the PEBP genes are mostly misannotated in the draft kiwifruit genome and the genome is generally poorly annotated, the mapping was performed on the 13 sequences representing the kiwifruit PEBP gene family, using STAR version 2.5.2a (Dobin *et al.*, 2013), and only uniquely mapped reads with no mismatches per paired alignment were used for gene expression studies. The gene expression unit was calculated as fragments per kilobase of transcript per million reads (FPKM).

### Yeast two-hybrid assay

*AcFT1* and *AcFT2* coding regions were recombined from entry clones into the GATEWAY destination vectors pDEST32 (pBDGAL4, bait) and pDEST22 (pADGAL4, prey) (Invitrogen). The remaining clones were as described before (Varkonyi-Gasic *et al.*, 2013). These vectors were individually introduced into *Saccharomyces cerevisiae* strains PJ69-4α (bait) and PJ69-4a (prey) (James *et al.*, 1996) for selection on minimal medium plates (WO) lacking leucine (bait) or trytophan (prey), followed by mating on YPAD plates and double sequential selection on WO medium lacking both leucine and trytophan. Final screening was performed on medium lacking trytophan, leucine, and histidine and supplemented with 0, 1, 2, 5, or 25 mM 3-amino-1,2,4-triazole. Plates were incubated for 4 d at 20 °C, photographed, and scored for growth. Reciprocal tests were performed in duplicate for all combinations.

## Results

### The *Actinidia* PEBP gene family

**Fig. 1.**
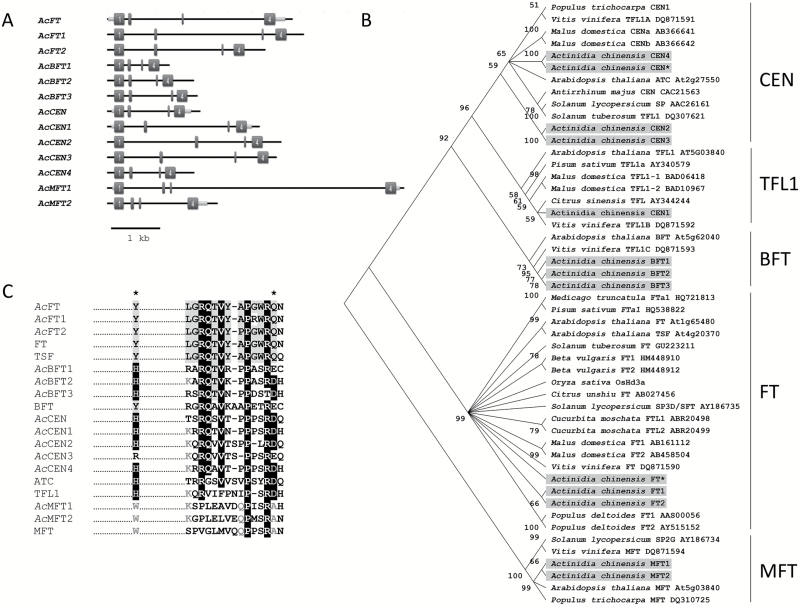
The *Actinidia* PEBP gene family. (A) Genomic organization of the genes. Dark gray boxes represent exons and light gray boxes represent untranslated region sequences (where available, based on RNA database searches). (B) Phylogram of plant PEBP proteins. The kiwifruit proteins are highlighted and previously described AcFT and AcCEN are indicated with an asterisk. (C) Partial amino acid alignment of *Actinidia* (*Ac*) and Arabidopsis PEBP proteins including the conserved segment B region.. Asterisks indicate Tyr85 (Y)/His88 (H) and Gln140 (Q)/Asp144 (D) residues distinguishing FT-like and TFL1-like members.

Searches for putative *FT*-, *TFL1/CEN*-, and *MFT*-like genes in the *A. chinensis* draft genome (Huang *et al.*, 2013), combined with analyses of the *Actinidia* EST database (Crowhurst *et al.*, 2008), revealed a total of 13 sequences representing the kiwifruit PEBP gene family (Fig. 1A). Five sequences with homology to *TFL1/CEN*, three with homology to related *BFT*, three with homology to *FT*, and two *MFT*-like sequences were identified, including previously characterized *Actinidia FT* (*AcFT*) and *CEN* (*AcCEN*) sequences (Varkonyi-Gasic *et al.*, 2013). Amplification and sequencing were further deployed to confirm full-length coding sequences. Predicted protein sequences were used for phylogenetic analysis (Fig. 1B) and for analysis of amino acid positions critical for function. Like the previously described AcFT, newly identified kiwifruit FT-like proteins, annotated AcFT1 and AcFT2, have the conserved glutamine within the external loop (14 amino acids in segment B) and the adjacent tyrosine residue, critical for FT function (Hanzawa *et al.*, 2005; Ahn *et al.*, 2006), but divergence in the external loop of AcFT1 (G137R) was identified (Fig. 1C). In addition, a change of isoleucine at the position highly conserved across the PEBP proteins (I117F) and cysteine at a position mostly conserved across FT-like proteins (C164G) was identified in AcFT1 (Supplementary Fig. S1). Additional divergent amino acids between AcFT1 and AcFT2 were at positions not highly conserved across FT proteins. With the exception of AcCEN3 and AcBFT1, all kiwifruit CEN- and BFT-like proteins have the conserved residues equivalent to Arabidopsis His88 and Asp144, while in the equivalent positions both MFT-like sequences have tryptophan and alanine residues (Fig. 1C).

### Differential expression of the *Actinidia TFL1/CEN*- and *BFT*-like genes

**Fig. 2.**
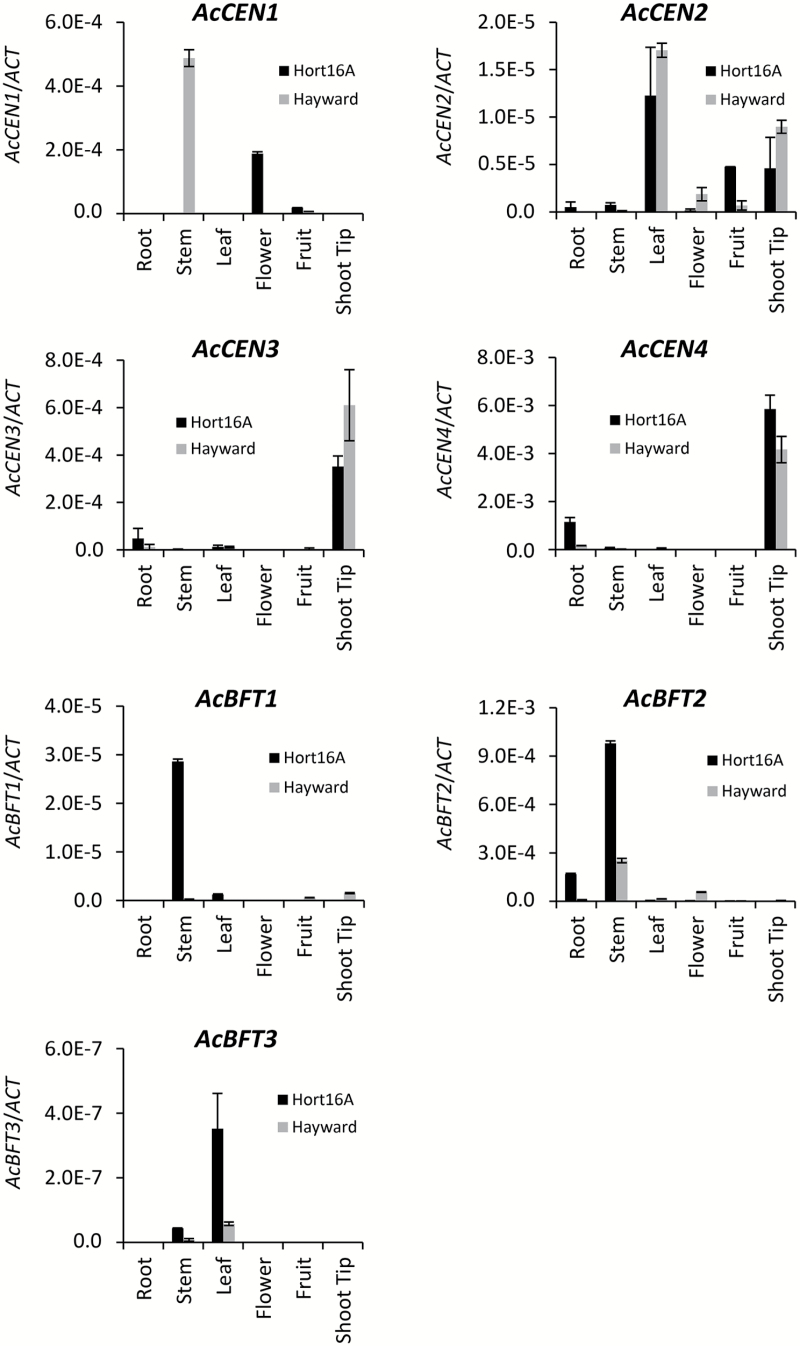
Relative expression ±SE of *Actinidia CEN*- and *BFT*-like genes in mature field-grown *A. chinensis* ‘Hort16A’ (black rectangles) and *A. deliciosa* ‘Hayward’ (gray rectangles) tissues, normalized against *ACTIN*.

Amplification of the full-length coding sequence of *Actinidia* PEBP genes often required cDNA from specific tissues (Supplementary Table S2), suggesting differential expression and mostly low expression levels. Real-time PCR amplification using gene-specific oligonucleotide primers was deployed to study organ-specific expression profiles further. In general, low expression was detected for all transcripts (Fig. 2). *AcCEN3* and A*cCEN4* transcripts were mostly confined to shoot tips, exhibiting a pattern similar to previously described *AcCEN* (Varkonyi-Gasic *et al.*, 2013). *AcCEN2* was detected in all samples, while *AcCEN1*, phylogenetically most similar to Arabidopsis *TFL1* (Fig. 1B), was absent from the shoot tip. All three *AcBFT* transcripts were amplified from the leaf sample, but *AcBFT2* was more abundant in the stem (Fig. 2).

### *Actinidia TFL1/CEN*- and *BFT*-like genes are functionally conserved floral repressors

**Fig. 3.**
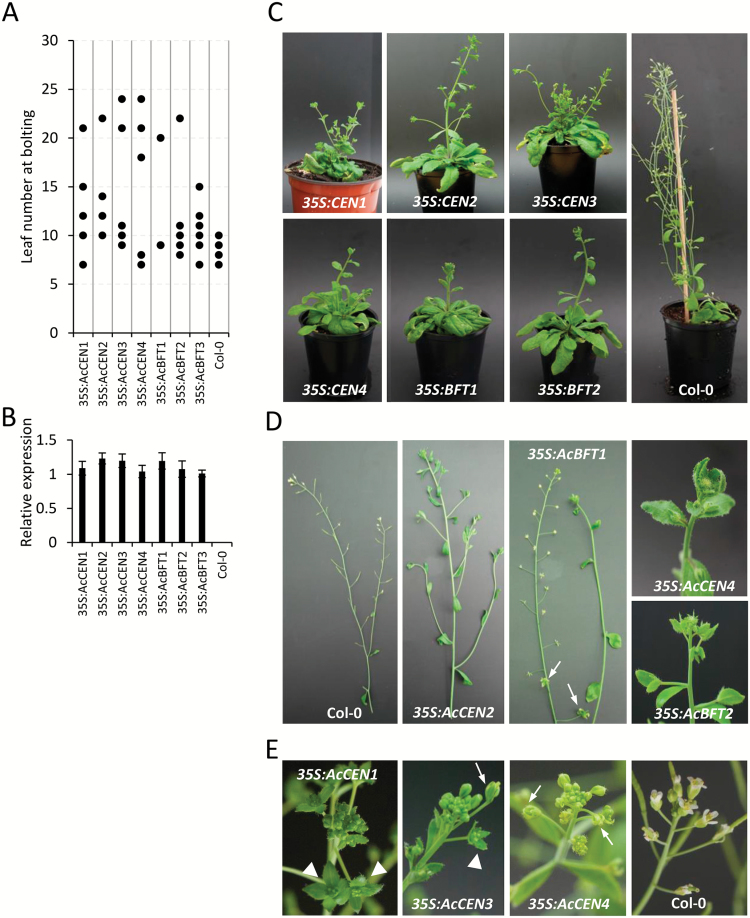
Constitutive expression of *Actinidia CEN*- and *BFT*-like genes affects flowering in Arabidopsis. (A) The range of flowering time recorded in T_1_ lines grown in inductive LD conditions. Five to eight lines per construct were monitored for flowering time, recorded as the number of rosette leaves when the primary inflorescence stems were 0.2 cm long. Each dot represents the flowering time of at least one primary transgenic line. (B) Transgene expression in representative late flowering lines, normalized to *ACT2*. (C) Representative late flowering plants, of the same age and grown under the same conditions as the control Col-0 plant. (D) Examples of altered inflorescence morphology: proliferation of coflorescences on a primary inflorescence stem of a *35S:AcCEN2* plant, leafy axillary shoot produced on a *35S:AcBFT1* plant with abnormal flowers on the primary inflorescence stem, and indeterminate inflorescence meristems of *35S:AcCEN4* and *35S:AcBFT2* plants that failed to produce flowers. Arrows indicate flowers with leaf-like sepals. (E) Examples of inflorescences with whorled phyllotaxis of leaf-like organs (arrowheads) and small abnormal flowers (arrows).

Typically, members of the PEBP family from various flowering plants have conserved roles in the regulation of flowering, so we elected to characterize the kiwifruit PEBP genes by constitutive expression in Arabidopsis. Coding sequences of *AcCEN1*, *AcCEN2*, *AcCEN3*, *AcCEN4*, *AcBFT1*, *AcBFT2*, and *AcBFT3* driven by the CaMV *35S* promoter were introduced into Arabidopsis Col-0, and a minimum of five kanamycin-resistant primary transgenic plants for each construct were monitored for flowering time in inductive LD conditions. Delayed flowering was observed with all constructs (Fig. 3A), which was correlated with high transgene expression levels (Fig. 3B). A severe delay in bolting combined with change in inflorescence architecture was demonstrated in at least one line for all constructs, with the exception of *AcBFT3* which resulted in moderately delayed bolting only. Transgenic plant phenotypes included large rosette leaves, highly branched leafy shoots resulting from proliferation of coflorescences in place of flowers on primary inflorescence stems, leafy axillary shoots developing from axillary meristems of rosette leaves, and third and fourth order branching from the first formed axillary shoots (Fig. 3C, D). The most severe impact was observed in *AcCEN4* and *AcBFT2* lines, which demonstrated prolonged indeterminacy of the inflorescence meristem (Fig. 3D), and some lines failed to produce flowers and siliques over the duration of the experiment. Development of inflorescences with whorled phyllotaxis of leaf-like organs and flowers with abnormalities such as reduced size, lack of petals, and leaf-like sepals were frequently found (Fig. 3E).

### *Actinidia MFT*-like genes have no impact on flowering time

**Fig. 4.**
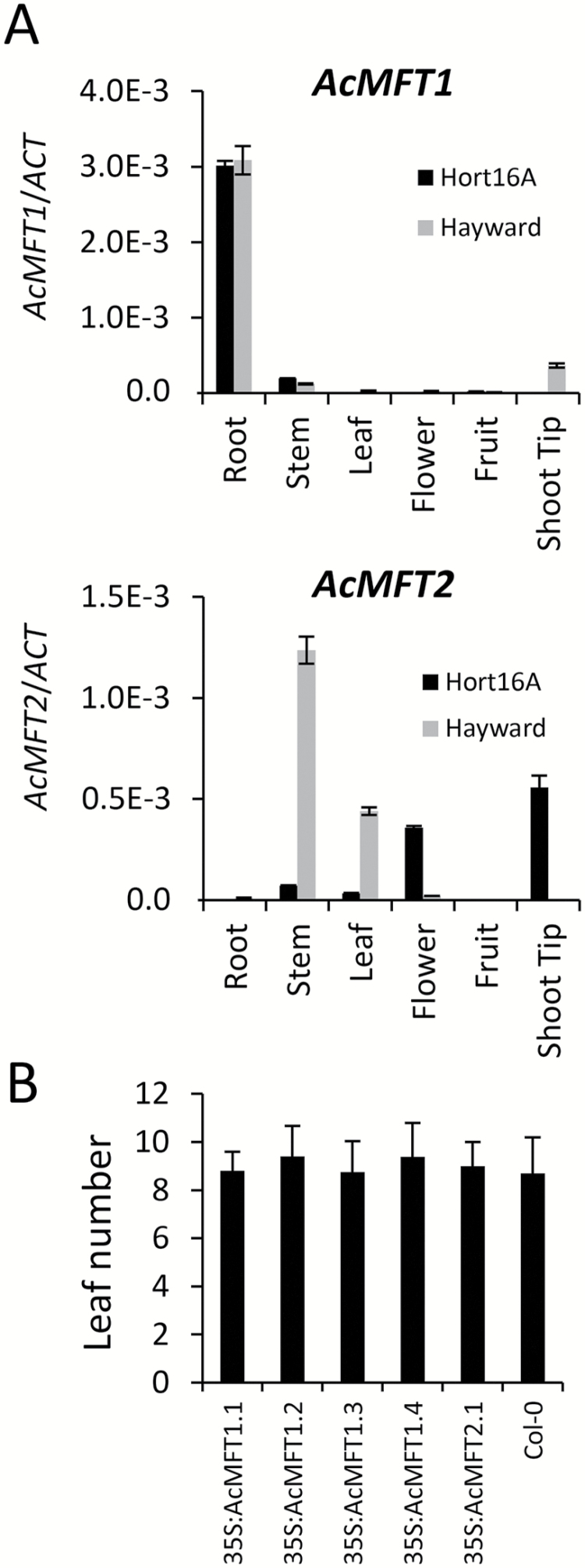
Characterization of *Actinidia MFT*-like genes. (A) Relative expression ±SE in *A. chinensis* ‘Hort16A’ (black rectangles) and *A. deliciosa* ‘Hayward’ (gray rectangles) tissues, normalized against *ACTIN*. (B) Wild-type flowering time in *35S:AcMFT* lines. Flowering time was recorded as the number of rosette leaves when the primary inflorescence stems were 0.2 cm long. Error bars represent the SD for 10 T_2_ transgenic lines grown in long-day (LD) conditions.

Arabidopsis *MFT* is highly expressed in developing seeds, but is also detected in root and leaf tissue (Xi *et al.*, 2010), and its constitutive expression led to slightly earlier flowering under LDs (Yoo *et al.*, 2004). *Actinidia MFT*-like transcripts were abundant in mature fruit and amplified from seed and flower tissue (Supplementary Table S2). Analysis using real-time PCR also identified expression in vegetative tissues for *AcMFT2* and particularly prominent root expression for *AcMFT1* (Fig. 4A), but very low expression was detected in axillary buds (Supplementary Table S3). To establish if kiwifruit *MFT*-like genes affect flowering time, coding sequences of *AcMFT1* and *AcMFT2,* each under the control of the *35S* promoter, were introduced into Arabidopsis Col-0. Twelve *AcMFT1* and four *AcMFT2* independent lines were generated and monitored for flowering time in non-inductive SD conditions. None of the plants bolted by the time they had the minimum of 30 rosette leaves. Wild-type flowering time was also observed in their progeny grown in LD conditions (Fig. 4B); therefore, we conclude that kiwifruit *MFT*-like genes do not promote flowering in transgenic Arabidopsis.

### Differential and largely opposing expression of *AcFT1* and *AcFT2*

**Fig. 5.**
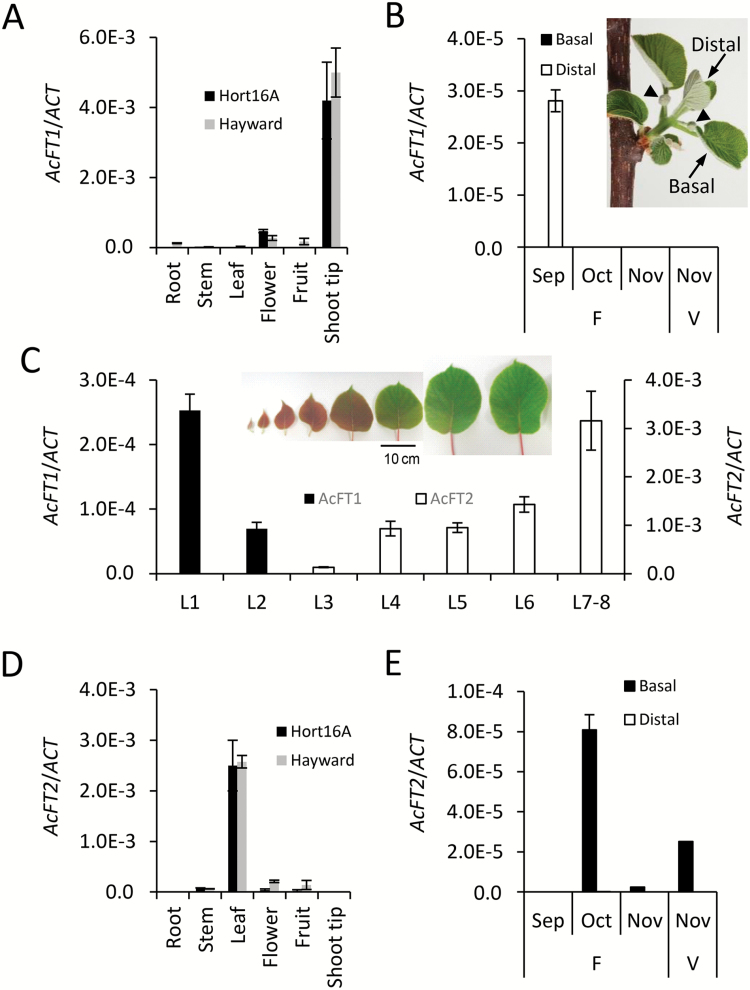
Expression patterns of *AcFT1* and *AcFT2* genes. (A) Relative *AcFT1* expression ±SE in *A. chinensis* ‘Hort16A’ (black rectangles) and *A. deliciosa* ‘Hayward’ (gray rectangles) tissues, normalized against *ACTIN*. (B) Relative *AcFT1* expression in basal and distal leaves of an *A. chinensis* ‘Hort16A’ growing shoots bearing developing flowers (F) and vegetative shoots (V) during the spring months. The insert shows morphology of a typical *A. chinensis* shoot in September. (C) Relative expression of *AcFT1* and *AcFT2* in the leaves of fast growing shoots collected from juvenile glasshouse-grown plants. The insert denotes L1–L8 leaf morphology. Because of the small size of L1 and L2 leaves, leaves collected from single shoots from three plants were pooled for RNA extraction. Another replicate of leaves from three single shoots is presented in Supplementary Fig. S2. (D) Relative *AcFT2* expression ±SE in *A. chinensis* ‘Hort16A’ (black rectangles) and *A. deliciosa* ‘Hayward’ (gray rectangles) tissues, normalized against *ACTIN*. (E) Relative *AcFT2* expression in basal and distal leaves of *A. chinensis* ‘Hort16A’ growing shoots bearing flowers (F) and vegetative shoots (V) during spring months. All sampling was performed at mid-day from at least three plants and the expression was normalized against *ACTIN*.

*FT*-like genes are of particular interest as key activators of flowering and so were studied in detail. *AcFT1* was preferentially expressed in terminal buds (Fig. 5A) and in the very small distal leaves during shoot outgrowth in early spring (Fig. 5B). Expression in the shoot tip and very small distal leaves was also observed in rapidly growing large shoots of young glasshouse-grown plants (Fig. 5C; Supplementary Fig. S2). In contrast, *AcFT2* was preferentially expressed in the leaf tissue (Fig. 5D). This expression was confined to large basal leaves of larger shoots, declined in mature leaves in late spring, and was detected on branches bearing flower buds, but also on vegetative shoots emerging after the first (floral) flush (Fig. 5E). *AcFT2* was also expressed in basal leaves of rapidly growing shoots of young glasshouse-grown plants, with a gradient of expression opposite to that of *AcFT1* (Fig. 5C; Supplementary Fig. S2).

### *AcFT1* and *AcFT2* are functionally conserved activators of flowering with differential impact on plant morphology

**Fig. 6.**
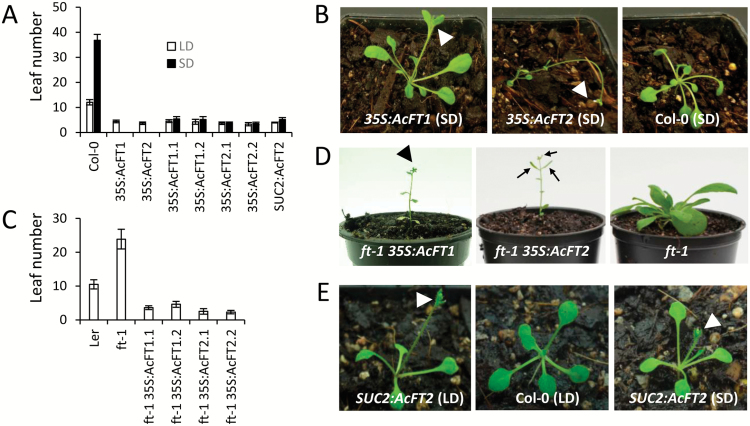
Expression of *AcFT1* and *AcFT2* genes promotes flowering in Arabidopsis. (A) Flowering time in inductive long-day (LD) and non-inductive short-day (SD) conditions. Flowering time was recorded as the number of rosette leaves when the primary inflorescence stems were 0.2 cm long. Error bars represent the standard deviation for 8 each *35S:AcFT1* and *35S:AcFT2* T_1_ transgenic lines grown in LD conditions, 10 progeny of two T_1_ lines for each construct grown in LD and SD conditions and 20 *SUC2:AcFT2* T_1_ transgenic lines grown in LD and SD conditions. (B) Differential impact of *35S:AcFT1* and *35S:AcFT2* on leaf size. Plants are of the same age and grown under the same conditions as the control Col-0 plant. (C) Flowering time of the Arabidopsis L*er ft-1* mutant in LD conditions. Flowering time was recorded as the number of rosette leaves when the primary inflorescence stems were 0.2 cm long. Error bars represent the standard deviation for 10 progeny of two T_1_ lines for each construct grown in LD conditions. (D) Representative early flowering *ft1* mutant plants expressing *AcFT1* and *AcFT2*. (E) Plants expressing *SUC2:AcFT2* flowered early in LD and SD conditions after normal leaf development. Arrowheads indicate flowers and arrows developing siliques.

To assess the potential of *AcFT1* and *AcFT2* to promote flowering, their coding sequences, driven by the *35S* promoter, were introduced into the Arabidopsis wild-type Col-0, and eight independent lines for each construct were monitored for flowering time. Early flowering was detected with all *AcFT1* and *AcFT2* plants in inductive LD conditions and with kanamycin-resistant progeny of two independent lines of each construct in LD and non-inductive SD conditions (Fig. 6A, B). Both constructs were evaluated for functional conservation in the L*er ft-1* late flowering mutant background. Both *35S:AcFT1* and *35S:AcFT2* complemented the *ft-1* mutation, and transgenic plants flowered much earlier than wild-type L*er* plants (Fig. 6C, D). To test the potential of *AcFT2* to perform the role of the leaf-derived phloem-mobile florigen, its coding sequence was placed under control of the vascular-specific *SUCROSE TRANSPORTER 2* (*SUC2*) promoter and introduced into transgenic Arabidopsis. The kanamycin-resistant lines demonstrated precocity in both day-length conditions (Fig. 6A, E). Similarly, *SUC2* promoter-driven *AcFT1* promoted flowering in transgenic Arabidopsis (Supplementary Fig. S3).

*35S:AcFT2* Arabidopsis lines typically flowered slightly earlier and often developed terminal flowers, very small leaves, and thin inflorescence stems, in contrast to mostly wild-type size leaves in early-flowering Col-0 *35S:AcFT1* lines (Fig. 6B). We hypothesized that AcFT1 divergence in one of the three previously identified positions (G137R in the external loop and I117F or C164G at positions mostly conserved across FT-like proteins; Supplementary Fig. S1) and altered binding to FD contribute to differential FT activity. However, similar phenotypes were observed when we introduced individual mutations at these positions in AcFT2 and reversed them in AcFT1, and lines with simultaneous mutations at all three positions in AcFT2 also produced relatively small rosette leaves (Supplementary Fig. S4). Similarly, both AcFT1 and AcFT2 were capable of interaction with Arabidopsis and kiwifruit FD proteins in a yeast-two hybrid assay, with similar interaction intensities (Supplementary Fig. S5).

### Constitutive expression of *AcFT1* and *AcFT2* leads to extreme precocity in transgenic kiwifruit

**Fig. 7.**
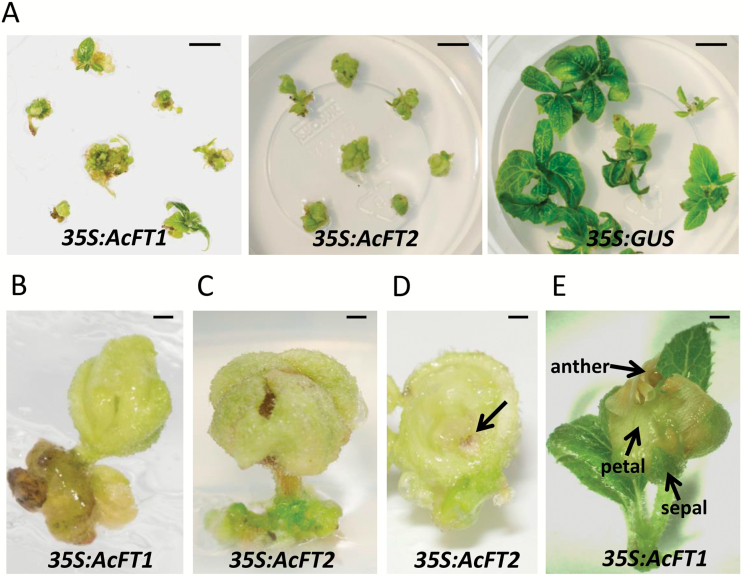
Transgenic kiwifruit flowering *in vitro.* (A) Flower and leaf, only flower, and only leaf develop in *35S:AcFT1*, *35S:AcFT2*, and control lines, respectively, 2 months after *Agrobacterium* inoculation. (B, C) *In vitro* flowers with undifferentiated floral organs. (D) Cross-section of an *in vitro* flower. The arrow indicates a rudimentary anther. (E) Leaves and differentiating floral organs in *35S:AcFT1* lines. Scale bars=10 mm (A) and 1 mm (B–E).

To test if *AcFT1* and *AcFT2* promote flowering and demonstrate differential activity in kiwifruit, their coding sequences driven by the *35S* promoter were introduced into *A. chinensis* using standard regeneration and transformation protocols. Major differences were observed 2 months after *Agrobacterium* inoculation; flower instead of leaf development was observed in the presence of ectopic *AcFT2*, but both leaf and flower development were recorded in the presence of ectopic *AcFT1* and only leaf development was recorded in the control (Fig. 7A). Both constructs gave rise to *in vitro* flowers (Fig. 7B, C), which aborted development soon after initiation of rudimentary reproductive organs (Fig. 7D), while some *AcFT1* lines developed leaves and flowers with differentiating floral organs (Fig. 7E).

### Seasonal expression patterns in axillary buds

**Fig. 8.**
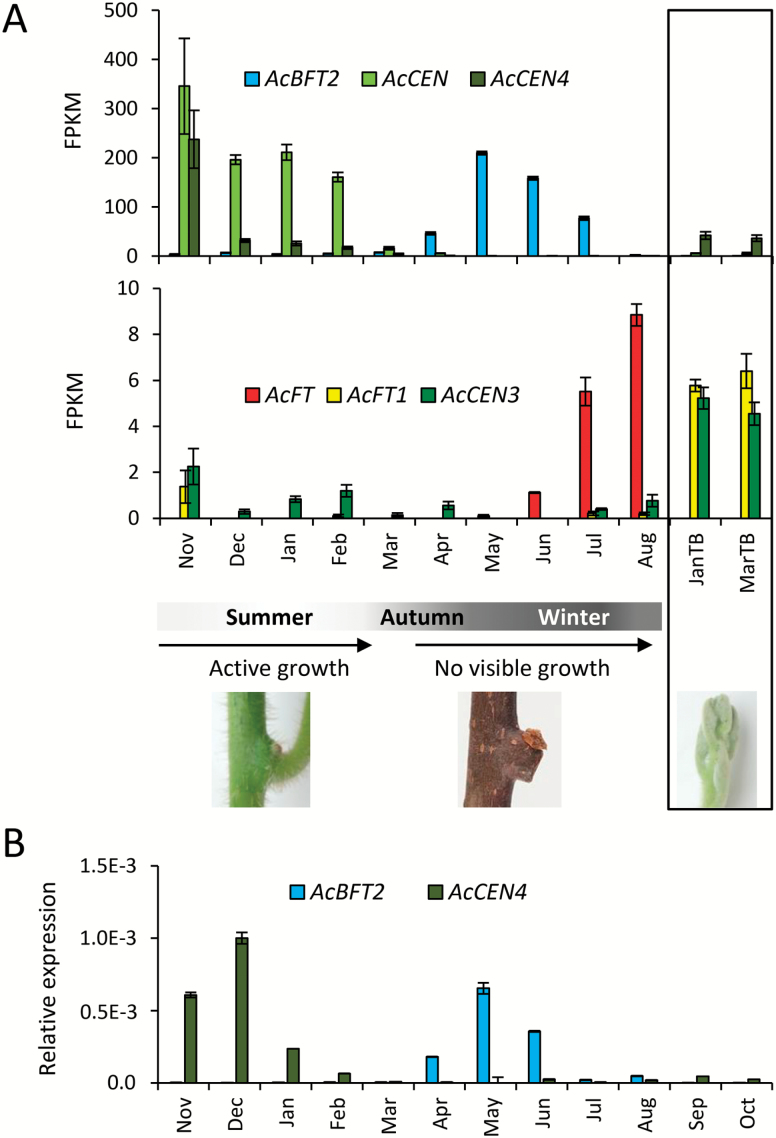
Expression in buds during the season. (A) *A. chinensis* axillary buds were collected at monthly intervals during the growth and dormancy cycle, and subjected to RNA-seq. Only uniquely mapped reads with no mismatches per paired alignment were used for gene expression studies. Frequency of gene expression was calculated as fragments per kilobase of transcript per million reads (FPKM) and is presented as the average ±SE of three biological replicates. The seasons and appearance of axillary buds are indicated below. Box, actively growing terminal buds were collected in January and March and subjected to RNA-seq as above. Frequency of gene expression is presented as the average ±SE of three and two biological replicates for January and March samples, respectively. The appearance of actively growing terminal buds is indicated below. (B) Relative expression ±SE in *A. deliciosa* axillary buds collected at monthly intervals during the growth and dormancy cycle, normalized against *ACTIN.* Expression of previously described genes is presented in Supplementary Fig. S6.

**Fig. 9.**
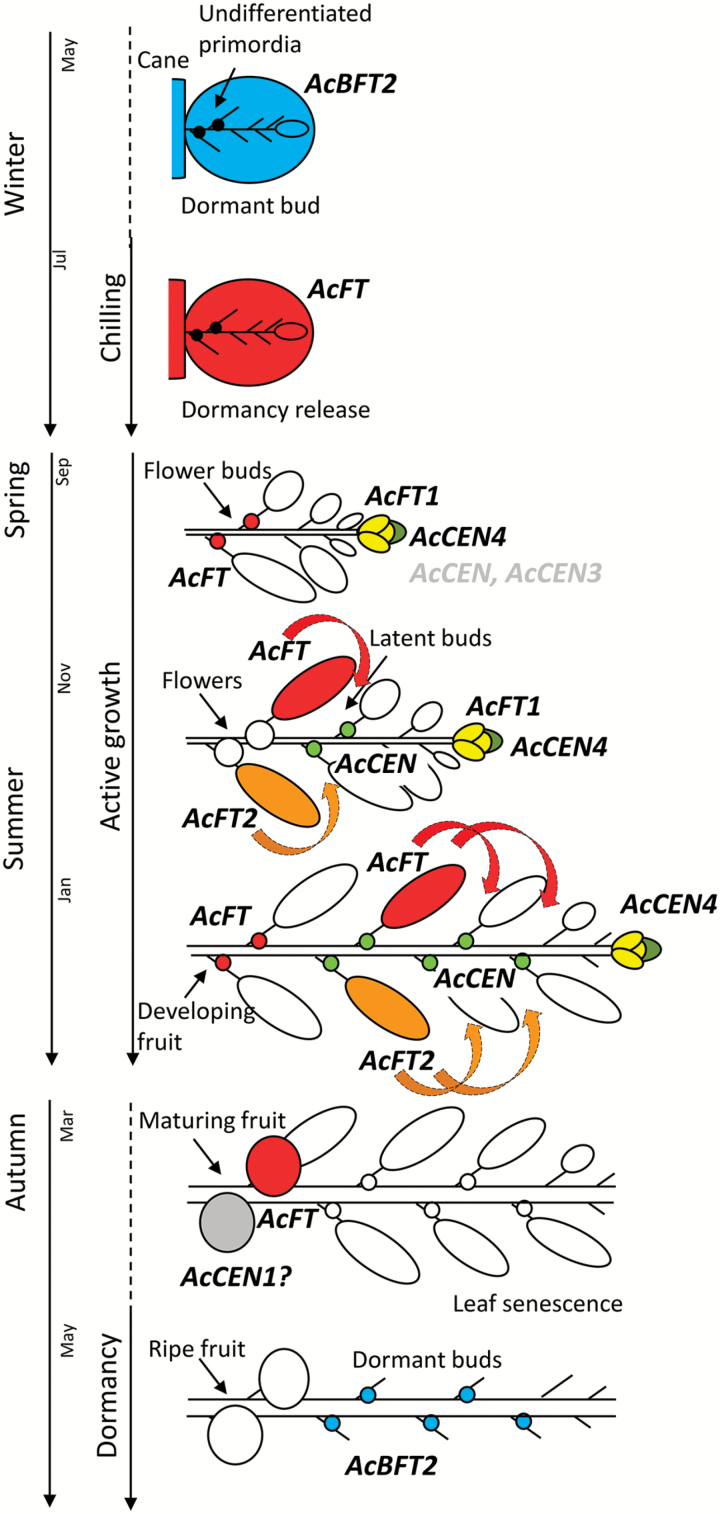
Schematic summarizing accumulation of transcripts in the shoot bud and developing shoot. *AcBFT2* (blue) accumulates at growth cessation and during dormancy in the bud. Undifferentiated meristems in the axils of leaf primordia are indicated by black dots. *AcFT* (red) accumulates with winter chilling and dormancy release. Shoot outgrowth is marked by expression of *AcCEN4* (dark green) and to a lesser degree *AcCEN* and *AcCEN3*, accompanied by accumulation of *AcFT1* (yellow). Subsequently, *AcFT2* (orange) accumulates in large, basal leaves. This expression is dynamic, declines with maturation, and is similar to that reported for *AcFT.* Dynamic accumulation of *AcFT* during flower and fruit development, but decline at maturation was also described (Varkonyi-Gasic *et al.*, 2013). *AcCEN1* (gray) is potentially associated with fruit development. *AcCEN* (light green) and to a lesser degree *AcCEN4* accumulate in developing (latent) buds during active shoot growth. Subsequently, *AcCEN* declines and *AcBFT* accumulates with the onset of dormancy. Potential FT protein movement is indicated by colored curved arrows.

To study the potential roles in dormancy, winter chilling, growth resumption, and initiation of flowering, expression of PEBP genes was further analyzed in kiwifruit axillary buds collected at regular intervals over the season. Interrogation of axillary bud transcriptomes identified differential expression of some *Actinidia CEN*- and *BFT*-like genes at different stages of bud development. *AcCEN* and *AcCEN4* were abundant in summer, during active growth, but gradually declined at the beginning of autumn, at which stage *AcBFT2* transcript abundance started to increase and remained high during the dormancy period. *AcCEN4* and to a lesser extent *AcCEN* and *AcCEN3* were also detected in actively growing terminal buds collected in summer and early autumn (Fig. 8A). Very low abundance or total absence of transcripts was observed for the remaining kiwifruit *CEN*- and *BFT*-like genes (Supplementary Table S3). Unlike *AcFT*, which was detected in buds during accumulation of winter chilling and growth resumption in spring, *AcFT1* and *AcFT2* transcripts were mainly absent from axillary buds and *AcFT1* was detected in samples of actively growing terminal buds collected in summer and early autumn (Fig. 8A). Accumulation of *AcBFT2* during establishment of dormancy was also detected in ‘Hayward’ axillary buds (Fig. 8B). Transcriptional activity of expressed PEBP genes is summarized in a schematic diagram (Fig. 9).

## Discussion

### The expansion and diversification of *Actinidia* floral repressor genes

Evolution of the PEBP family has had an important role in plant diversification and adaptation. *Actinidia* have undergone two recent whole-genome duplication events, which followed the ancient hexaploidization event shared by core eudicots (Huang *et al.*, 2013). As a result of these events, the *Actinidia* PEBP gene family contains multiple *FT*-, *CEN*-, and *BFT*-like genes. The expanded family of floral repressors in kiwifruit might be of significant relevance to plant growth, architecture, and flowering. All kiwifruit *CEN*- and *BFT*-like genes drive indeterminacy and delay flowering in Arabidopsis, and at least one of them, *AcCEN*, inhibits flowering in *Actinidia eriantha* (Varkonyi-Gasic *et al.*, 2013). Together with *AcCEN*, *AcCEN3* and *AcCEN4* appear to be preferentially expressed in the buds and are thus likely to perform a role in regulation of flowering, architecture, and determinacy as functional homologs of Arabidopsis *TFL1*. Determinacy is an agronomically important trait associated with domestication, and homologs of *TFL1/CEN* genes have been implicated in agricultural adaptation in crops such as tomato, barley, grapevine, bean, soybean, and strawberry (Pnueli *et al.*, 1998; Liu *et al.*, 2010; Fernandez *et al.*, 2010; Tian *et al.*, 2010; Comadran *et al.*, 2012; Iwata *et al.*, 2012; Koskela *et al.*, 2012; Repinski *et al.*, 2012). Kiwifruit has been developed into a horticultural crop only recently (Ferguson, 1990). Most cultivars are either direct selections from the wild or are only a few generations removed from the wild, with only a short history of systematic breeding (Datson and Ferguson, 2011). Currently, excessive vegetative vigor is managed through costly manual pruning. For that reason, combined with generally low harvest index of current kiwifruit cultivars, manipulation of the shoot architecture remains a significant breeding target. Therefore, *AcCEN*, *AcCEN4*, and *AcCEN3* represent prime candidates for kiwifruit improvement.

In contrast to kiwifruit *CEN*-like transcripts, which were associated with active growth and declined before the establishment of dormancy, expression of a member of the kiwifruit *BFT* clade is highest during winter dormancy and peaks at the time of leaf drop. The role of BFT proteins remains poorly understood. Arabidopsis BFT has a TFL1-like activity (Yoo *et al.*, 2010) and is associated with stress response (Ryu *et al.*, 2011, 2014). Up-regulation of Arabidopsis *BFT* following abscisic acid (ABA), drought, or osmotic stress treatment (Chung *et al.*, 2010) resembles *AcBFT2* transcript accumulation during winter dormancy, a process which is also potentially mediated by ABA and involves cold-, drought-, and stress-regulated genes (Horvath *et al.*, 2008). In Arabidopsis, high *BFT* expression causes suppression of axillary inflorescence development (Yoo *et al.*, 2010). In kiwifruit, *AcBFT2* transcript accumulation is associated with suppression of latent bud growth during unfavorable winter conditions. Thus, *AcBFT2* may repress vegetative growth, but could also maintain the undifferentiated state of meristems in the axils of leaf primordia in the dormant bud (Fig. 9), which develop as inflorescences after sufficient chilling. Interestingly, apple *MdBFTa* and *MdBFTb* were expressed at higher levels in dwarfing rootstocks, commonly used to reduce the scion vigor, suggesting a conserved role in suppression of axillary shoot development (Foster *et al.*, 2013). Similarly, expression of a grapevine *BFT* homolog, *VvTFL1C*, increased progressively during latent bud development (Carmona *et al.*, 2007), suggesting that aspects of BFT function may be conserved in woody perennials.

*AcCEN1*, phylogenetically closest to *TFL1*, and *AcCEN2* appear to be expressed in a range of tissues, including the fruit. The relevance of this is unclear at this stage, but is in line with reports of *TFL1/CEN*-like gene expression in fleshy fruit (Lindqvist *et al.*, 2006; Mimida *et al.*, 2009; Li *et al.*, 2014). The current study provides no evidence that *MFT*-like genes contribute towards regulation of flowering and architecture in kiwifruit. Their expression in seed may be similar to a proposed role in hormonal signaling regulating seed germination in Arabidopsis (Xi *et al.*, 2010; Footitt *et al.*, 2011) and wheat (Nakamura *et al.*, 2011), but the universality of this *MFT*-like role is yet to be confirmed in kiwifruit.

### The diversification of *Actinidia FT* genes

The *FT*-like genes have emerged with the evolution of the angiosperms, consistent with their key role in regulation of flowering (Shalit *et al.*, 2009; Karlgren *et al.*, 2011), while subsequent functional divergence contributed to the redundant, similar, or novel, and in some cases antagonistic *FT* functions (Blackman *et al.*, 2010; Pin *et al.*, 2010; Hsu *et al.*, 2011;Navarro, 2011; Harig *et al.*, 2012; Lee *et al.*, 2013; Cao *et al.*, 2016). The three *Actinidia FT* genes probably reflect two recent whole-genome duplication events in kiwifruit (Huang *et al.*, 2013), after which these genes have evolved divergent expression patterns. The previously described *AcFT* gene expression in response to cold correlated with winter chilling requirement and bloom time of kiwifruit cultivars, but it failed to promote flowering in kiwifruit (Varkonyi-Gasic *et al.*, 2013). This expression at later stages of dormancy coincided with reduction in *AcBFT2* transcript accumulation, suggesting that antagonism between these two genes may be of importance for bud dormancy and growth cycles.

In contrast, *AcFT1* and *AcFT2* both act as potent activators of flowering. This function is conserved across diverse plant species and may be non-cell autonomous, as demonstrated for AcFT2, thus fulfilling the criteria for florigen (Chailakhyan, 1968). The severity of phenotypes in both Arabidopsis and kiwifruit plants constitutively expressing high levels of *AcFT2* suggests that it may act in a dosage-dependent manner across long distances, when high expression may provide sufficient amounts to be delivered to the meristems in latent buds, to balance out the repressors of flowering and establish floral fate. Conversely, *AcFT1* expression differs from the florigen concept in that its expression appears restricted to kiwifruit sink tissue. Therefore, it might be fulfilling a role other than flowering control, perhaps in regulation of terminal bud expansion rate and abortion, which underlies high developmental plasticity of kiwifruit (Foster *et al.*, 2007). Alternatively, both AcFT1 and AcFT2 may act locally; in particular, AcFT2 may have a role in termination of leaf expansion and development, while AcFT1 may regulate termination of the shoot apical meristem after establishment of leaf fate.

Comparison of amino acid sequences identified divergence of AcFT1 residues in positions highly conserved in other FT-like proteins, which did not abolish its function and had no effect on the interaction with FD proteins, further confirming the robust nature of FT proteins as activators of the flowering process. The substitution for polar-positive arginine in the position of non-polar-neutral glycine in the conserved segment B (G137R) had little effect on AcFT1 activity, as recently demonstrated in Arabidopsis (Ho and Weigel, 2014) and consistent with findings in sugar beet (Klintenäs *et al.*, 2012). A conservative I117F substitution did not affect FT function, despite conservation of isoleucine in this position in FT and most other PEBP proteins (Supplementary Fig. S1) (Ho and Weigel, 2014). Similarly, introduction of a glycine in the position of highly conserved cysteine (C164G) had no impact on AcFT activity. Therefore, the sum of differences in these and other positions and interactions with proteins other than FD, including TEOSINTE-BRANCHED1/CYCLOIDEA/PCF (TCP) transcription factors (Mimida *et al.*, 2011; Niwa *et al.*, 2013; Ho and Weigel, 2014), and binding of unknown ligand(s) might provide the basis for different flowering time and architecture of *35S:AcFT1* and *35S:AcFT2* lines.

In summary, multiple differentially expressed and functionally divergent *FT*- and *CEN*-like genes regulate flowering and architecture in kiwifruit, and a *BFT*-like gene is associated with a lack of growth during dormancy. Specific expression of *FT*-like activators and *CEN*/*BFT*-like repressors (Fig. 9) may ensure adequate timing of growth and dormancy, leaf, flower, and fruit development, shoot tip growth, and establishment of latent buds. Manipulation of their dosage and functionality may provide practical means to reduce juvenility, hasten the breeding process, improve orchard management, and increase kiwifruit productivity.

## Supplementary Material

Supplementary Data
