# Supplementary material for: Three FT and multiple CEN and BFT genes regulate maturity, flowering, and vegetative phenology in kiwifruit
Source: J Exp Bot. 2017 Mar 27;68(7):1539–53. doi: 10.1093/jxb/erx044 (PMC5441913; doi:10.1093/jxb/erx044)
Supplement: Supplementary Data [file erx044_Supplementary_Data.zip › Supplementary_Figures_S1_S6.pdf]

Supplementary Figure S1

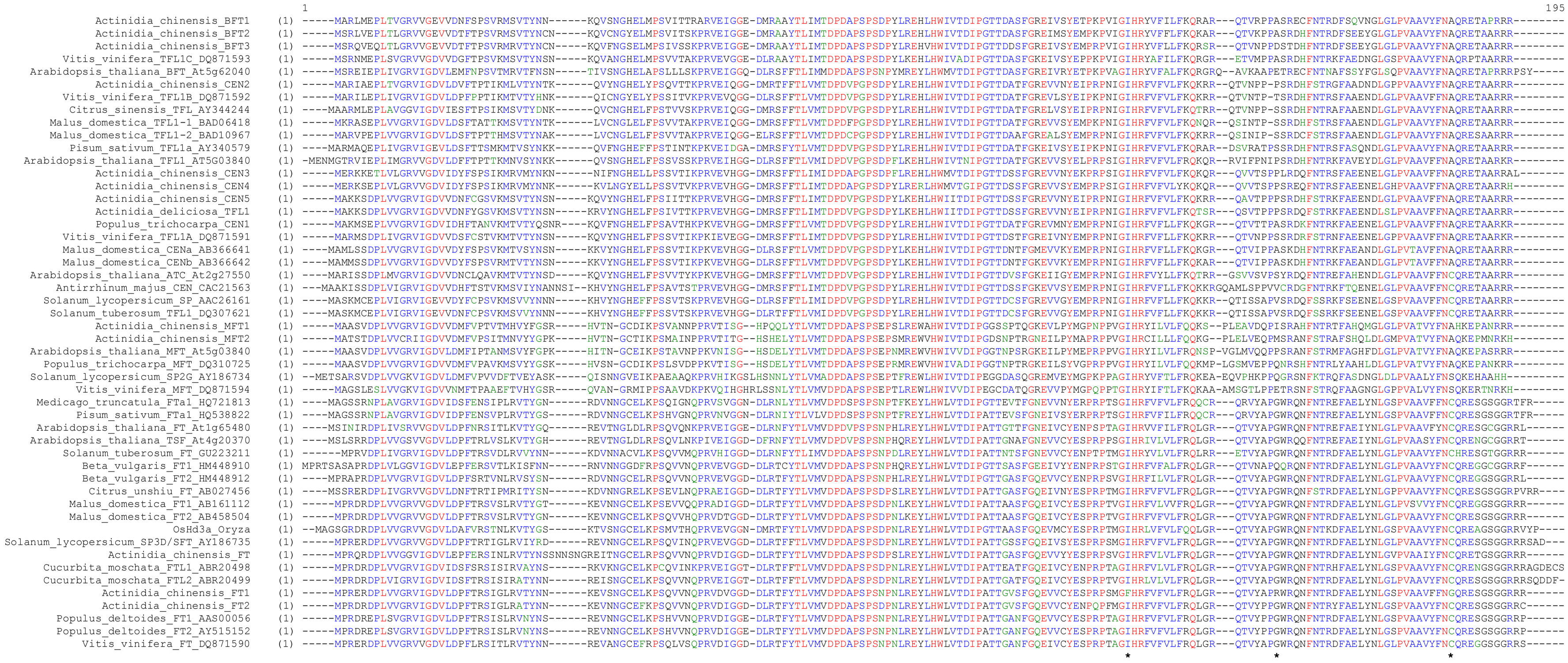

Fig. S1. Alignment of PEBP protein sequences from a range of plant species. AcFT1 divergence is indicated by asterisks.

Supplementary Figure S2

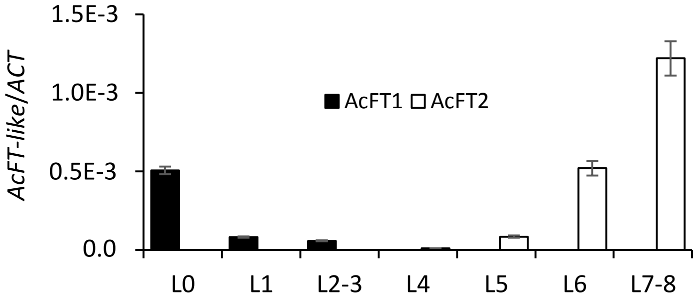

Fig. S2. Relative expression of *AcFT1* and *AcFT2* in the leaves of fast growing shoots collected from juvenile glasshouse-grown plants. Sampling was performed from three plants and leaves of similar size were pooled for RNA extraction. L0, shoot tip. Leaf size and maturity is increasing from L1 to L8.

Supplementary Figure S3

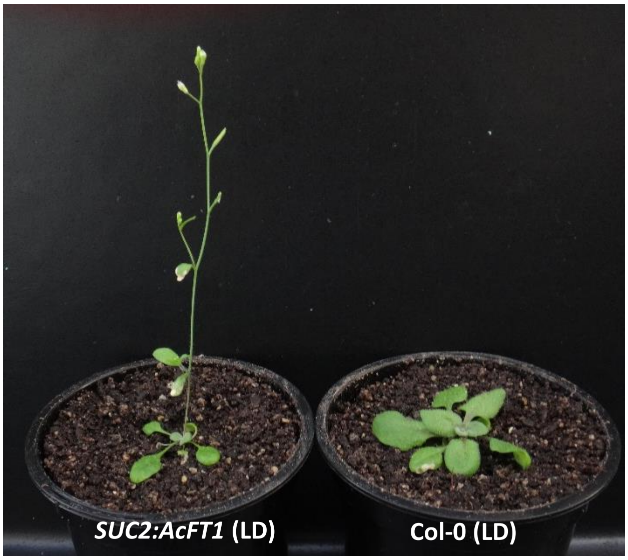

Fig. S3. Representative early flowering *SUC2:AcFT1* Arabidopsis plant, of the same age and grown under the same LD conditions as the control Col-0 plant.

Supplementary Figure S4

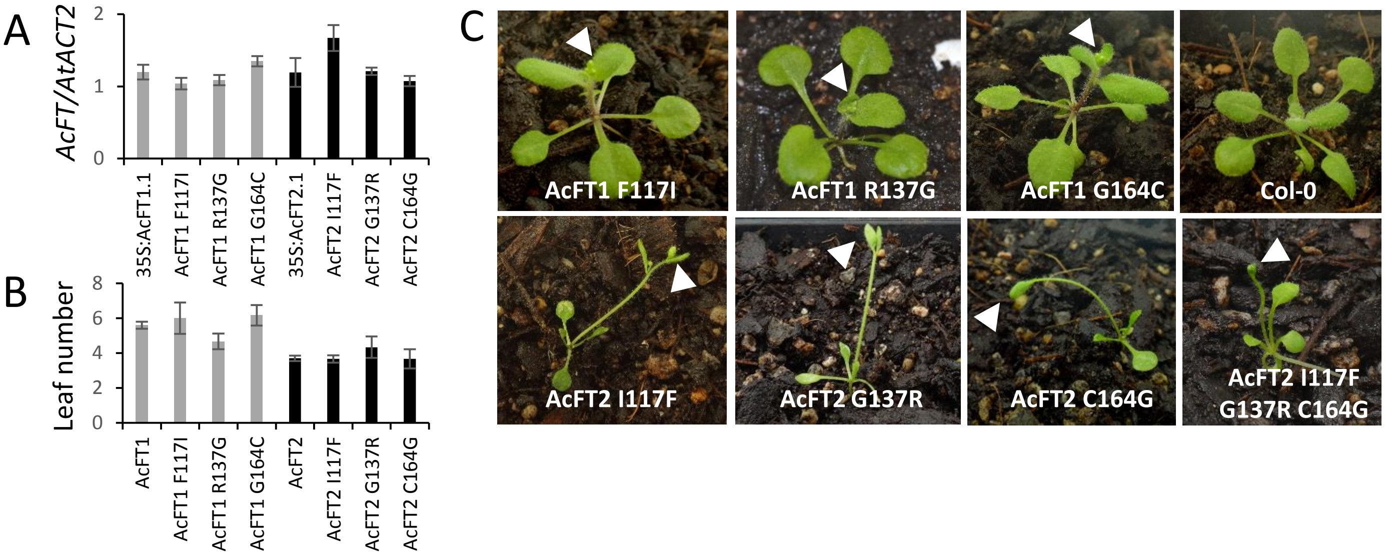

Fig. S4. Mutagenesis of divergent AcFT1 residues had little impact on flowering time and leaf size. (a) Relative transgene expression ± SE in representative lines. (b) Flowering time recorded as the number of rosette leaves ± SE for the minimum of six lines per construct, grown in short day (SD) conditions. Grey bars, AcFT1 mutagenesis. Black bars, AcFT2 mutagenesis. (c) Representative plants with indicated mutations. All plants are of the same age and grown under the SD conditions as the representative control Col-0 plant. Arrowheads indicate flowers. Amino acid positions refer to the Arabidopsis FT positions.

Supplementary Figure S5

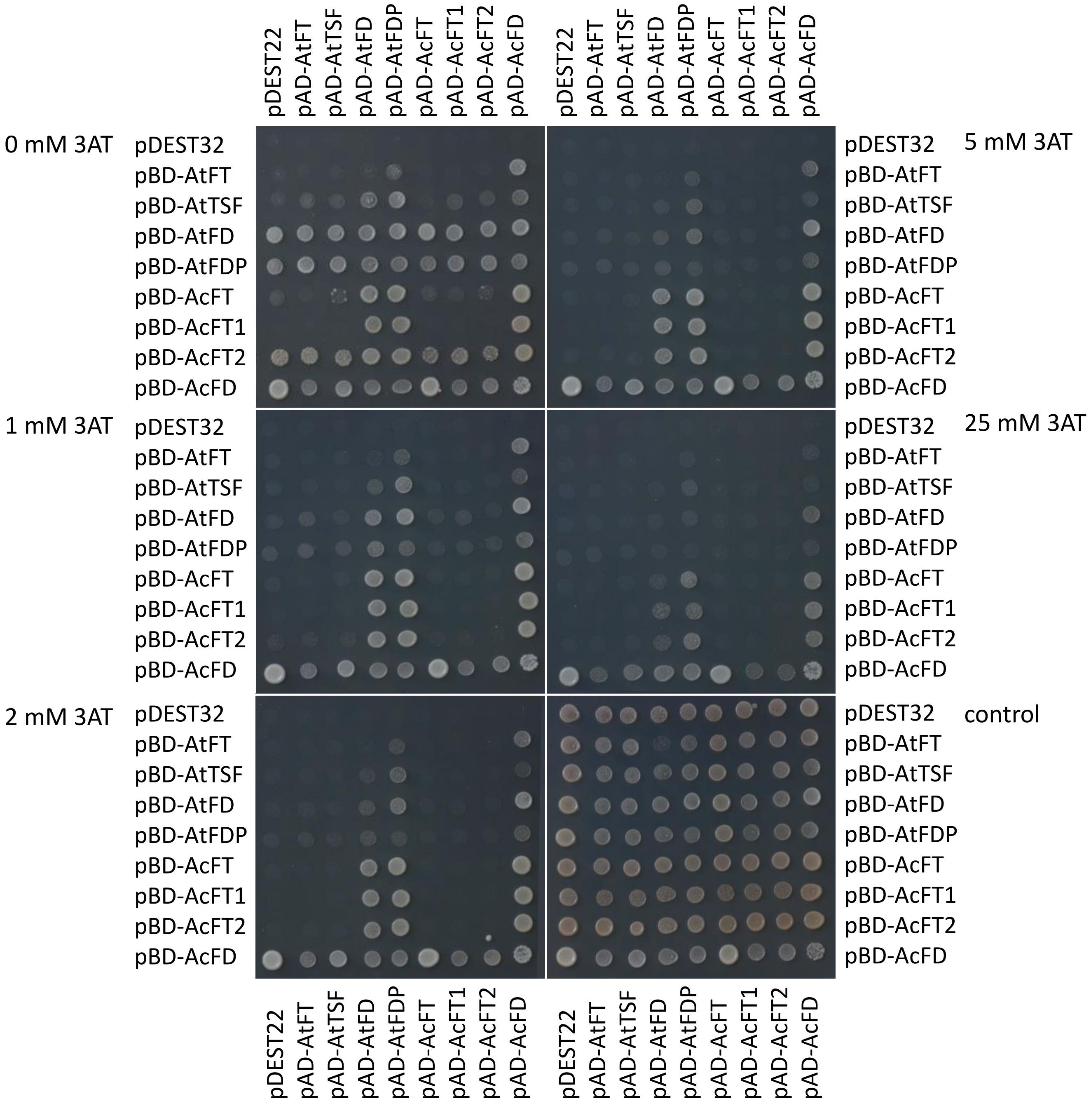

Fig. S5. Yeast-two-hybrid assays were used to evaluate interactions between FT and FD proteins Ac, *Actinidia chinensis*. At, Arabidopsis.3AT, 3-amino-1,2,4-triazole.

Supplementary Figure S6

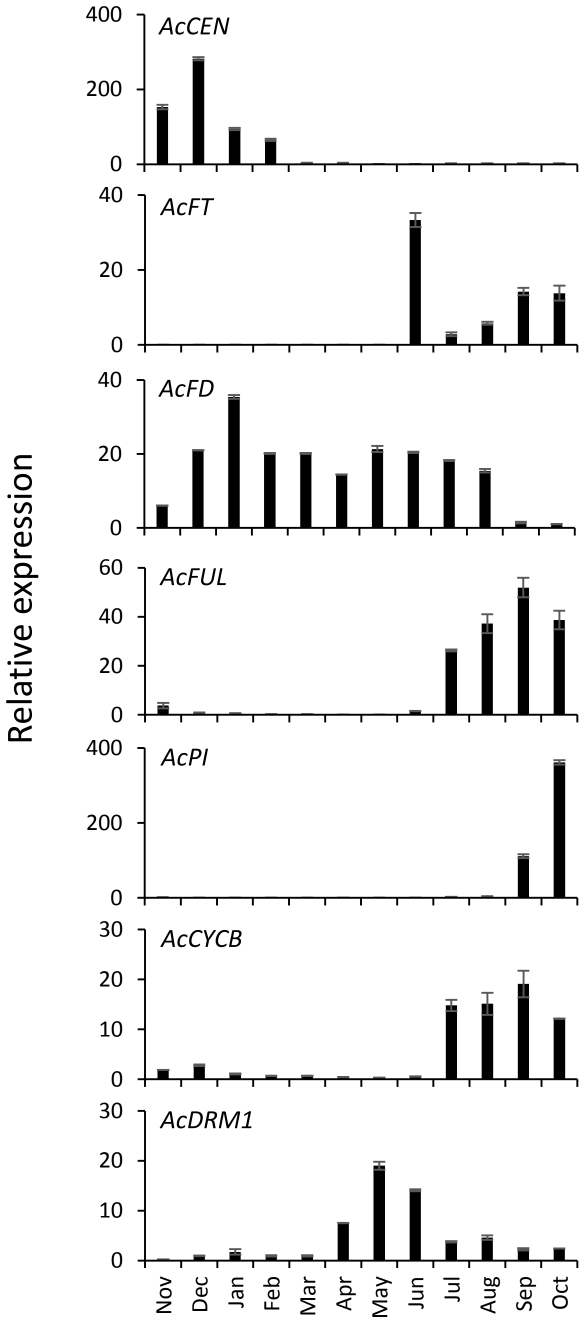

Fig. S6. Relative expression ± SE in *A. deliciosa* axillary buds collected at monthly intervals during the growth and dormancy cycle. Expression was normalized against *ACTIN* and expressed as fold change to one of the time points. The genes and oligonucleotide primers have been described (Varkonyi-Gasic *et al.* 2013; Varkonyi-Gasic *et al.* 2011). *Actinidia CYCLIN B* (*AcCYCB*) and *DORMANCY-ASSOCIATED PROTEIN 1* (*AcDRM1*) were used as markers for cell division and dormancy, respectively. *AcPI* (kiwifruit *PISTALLATA*) marks floral organ development.
